# [Corrigendum] Ginsenoside Rg1 ameliorates apoptosis, senescence and oxidative stress in ox-LDL-induced vascular endothelial cells via the AMPK/SIRT3/p53 signaling pathway 

**DOI:** 10.3892/etm.2026.13301

**Published:** 2026-09-16

**Authors:** Tian-Jiao Lyu, Zi-Xiu Zhang, Jun Chen, Zong-Jun Liu

Exp Ther Med 24:545, 2022; DOI: 10.3892/etm.2022.11482

Following the publication of the above article, a concerned reader drew to the Editor’s attention that one of the antibodies the authors had used in their study was apparently selected inappropriately: The antibody (cat. no. ab51243) that the authors reported to have used is specific for the protein known as p16-ARC (or ARPC5), not the apoptosis-associated protein p16-INK4a, as was intended.

Upon re-examining reagent records, the authors identified an annotation issue regarding the anti-p16 antibody (cat. no. ab51243) used for western blot detection. This antibody was intended to detect p16-INK4a (encoded by *CDKN2A*); however, as noted above, this antibody targets p16-ARC (encoded by ARPC5), a distinct protein with a similar name. Therefore, all original western blot data labelled p16-INK4a in this paper are invalid. However, the authors wished to point out that their conclusions regarding endothelial senescence in this study were supported by multiple independent valid datasets obtained from well-validated assays: SA-β-gal senescence staining, p21 western blotting, along with the expression analysis of AMPK, SIRT3 and p53. These measurements constituted the primary evidence supporting the proposed mechanism linked to the AMPK/SIRT3/p53 signalling axis. In addition, previously published studies utilizing the ox-LDL-treated HUVEC model (for example, the paper by Gerard Karsenty and colleagues entitled “Inhibition of leptin regulation of parasympathetic signaling as a cause of extreme body weight-associated asthma.” Cell Metab. vol. 17.1 (2013); p. 35-48) have demonstrated that cellular senescence and its underlying mechanisms can be adequately characterized by senescence staining combined with p21- and p53-associated signalling measurements. In view of this, the authors considered that removing the p16 data from the paper would not compromise the conclusions drawn in this study. All other experimental datasets, including SA-β-gal senescence staining, p21 western blotting, apoptosis detection, oxidative stress measurement and AMPK/SIRT3/p53 pathway analysis, were generated with fully validated reagents, and remain scientifically valid.

After considering this appeal, the Editor has approved the authors’ request that the p16 data be omitted from this study. Therefore, all references to “p16” in this study (as were featured in the Materials and methods, Results and Discussion sections) should be considered to be eliminated from this paper. Revised versions of Fig. 3 and 7 have been provided, with the p16 data relating to the western blot analyses removed from Fig. 3C and 7C respectively, and these are featured on the next two pages. Revised figure legends, omitting all references to p16, have also been provided.

Note that the omission of the data relating to p16 does not affect the overall conclusions reported in the paper. The authors express their thanks to the Editor of *Experimental and Therapeutic Medicine* for allowing them the opportunity to publish this corrigendum. All the authors have agreed to the publication of this corrigendum, and wish to apologize to the readership for any inconvenience caused.

## Figures and Tables

**Figure 3:**
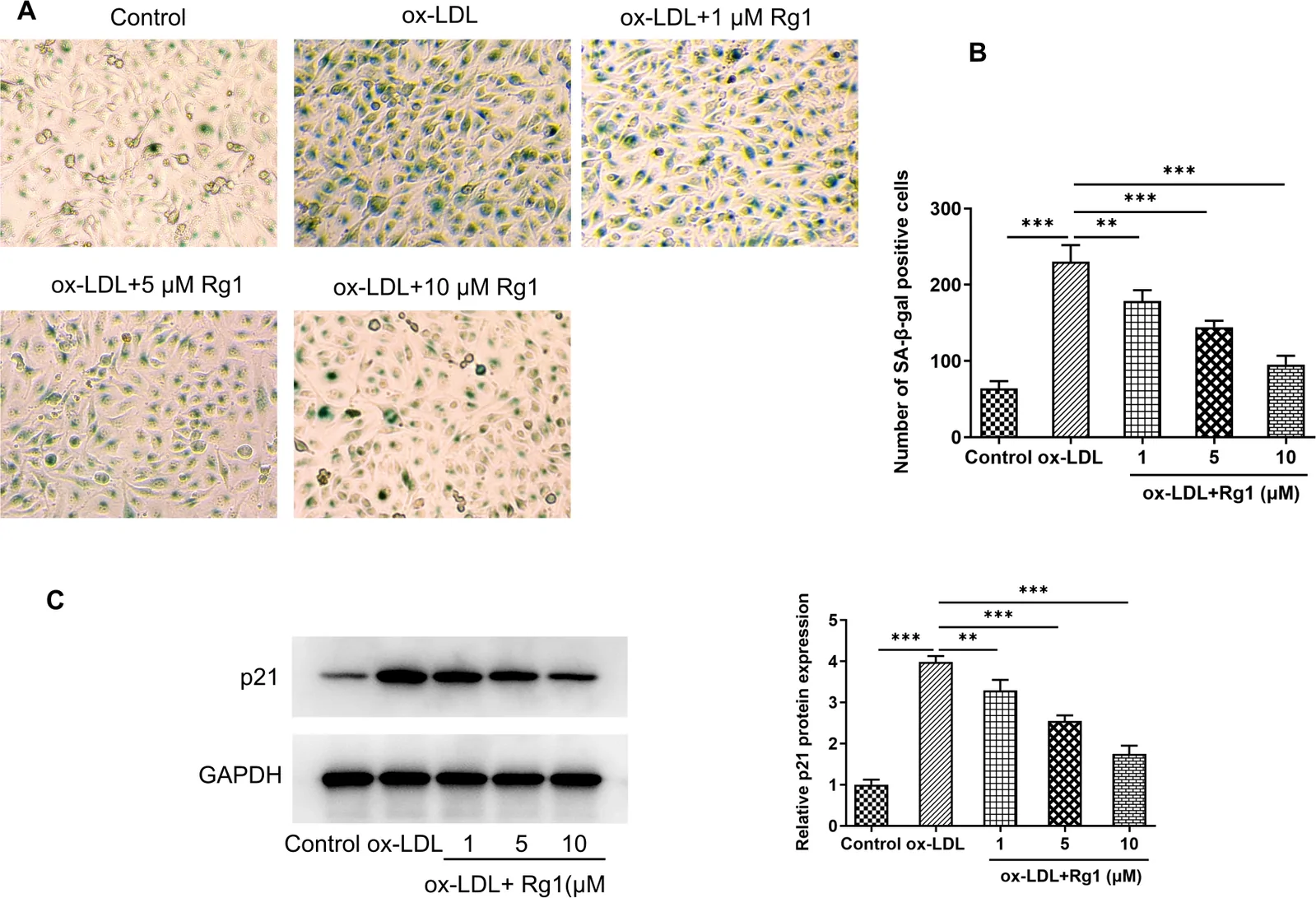
Rg1 inhibits the senescence of ox-LDL-induced HUVECs. (A) SA-β-gal staining was used to detect the senescence of ox-LDL-induced HUVECs (magnification, ×200). (B) Quantified number of senescent cells. (C) Western blot analysis was used to detect the expression of senescence-related protein p21. ^**^P<0.01, ^***^P<0.001. ox-LDL, oxidized low-density lipoprotein; HUVEC, human umbilical vein endothelial cell; Rg1, ginsenoside Rg1; SA-β-gal, senescence-associated β-galactosidase.

**Figure 7:**
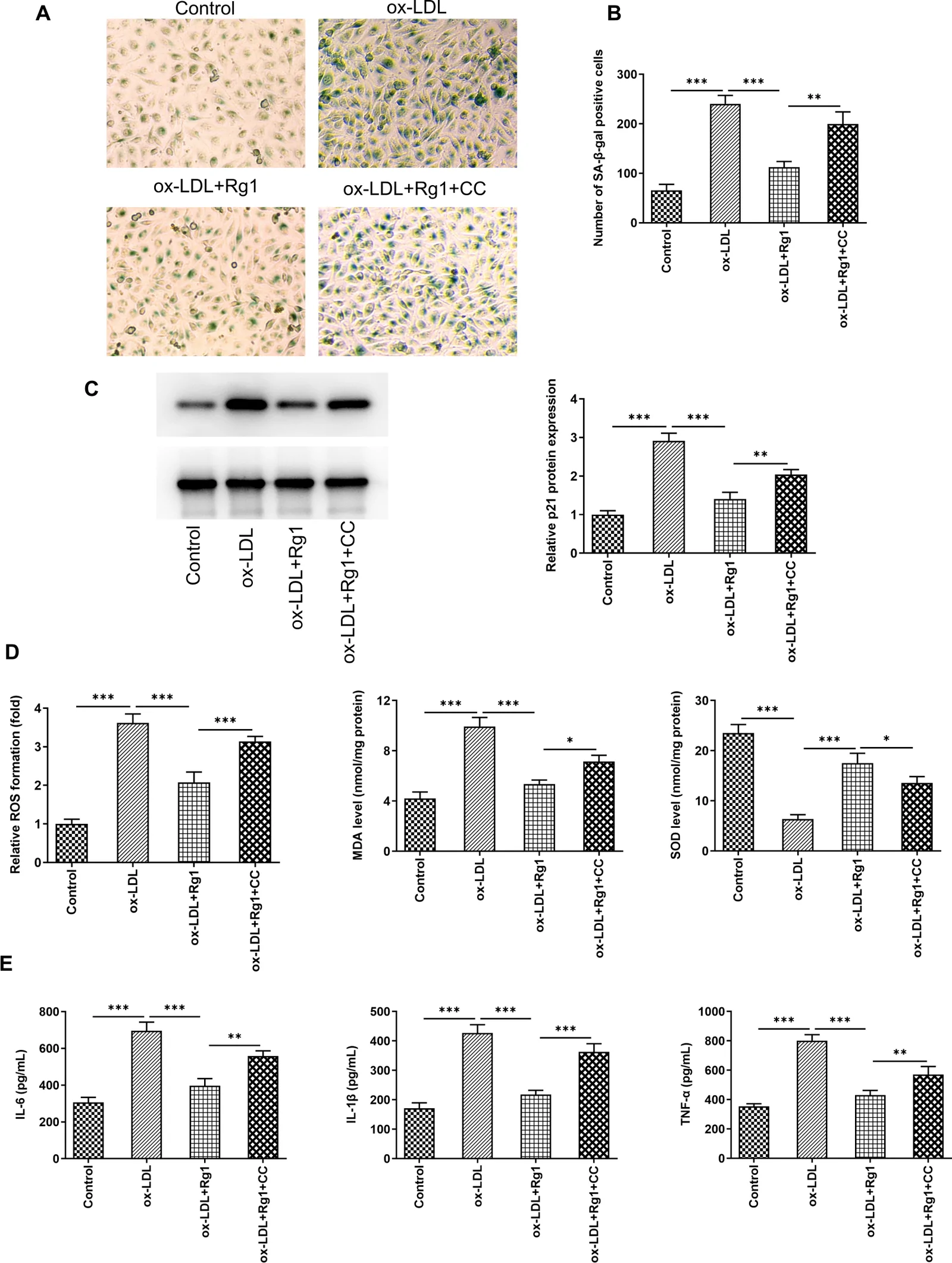
Pretreatment with CC partially reverses the protective effect of Rg1 on ox-LDL-induced HUVECs. (A) SA-β-gal staining was used to detect the senescence of HUVECs (magnification, ×200). (B) Quantified number of senescent cells. (C) Western blot analysis was used to detect the expression of senescence-related protein p21. (D) Related kits were used to detect the expression of oxidative stress indicators. (E) ELISA was performed to detect the release of inflammatory cytokines. ^*^P<0.05, ^**^P<0.01, ^***^P<0.001. ox-LDL, oxidized low-density lipoprotein; HUVEC, human umbilical vein endothelial cell; Rg1, ginsenoside Rg1; CC, compound C; SA-β-gal, senescence-associated β-galactosidase.

